# Predicting three-dimensional chaotic systems with four qubit quantum systems

**DOI:** 10.1038/s41598-025-87768-0

**Published:** 2025-02-20

**Authors:** Joel Steinegger, Christoph Räth

**Affiliations:** 1 Institut für KI Sicherheit, Deutsches Zentrum für Luft- und Raumfahrt (DLR), Wilhelm-Runge-Straße 10, 89081 Ulm, Germany; 2 Institut für Materialphysik im Weltraum, Deutsches Zentrum für Luft- und Raumfahrt (DLR), Linder Höhe, 51170 Köln, Germany

**Keywords:** Computational science, Quantum physics, Information theory and computation

## Abstract

Reservoir computing (RC) is among the most promising approaches for AI-based prediction models of complex systems. It combines superior prediction performance with very low CPU-needs for training. Recent results demonstrated that quantum systems are also well-suited as reservoirs in RC. Due to the exponential growth of the Hilbert space dimension obtained by increasing the number of quantum elements small quantum systems are already sufficient for time series prediction. Here, we demonstrate that three-dimensional systems can already well be predicted by quantum reservoir computing with a quantum reservoir consisting of the minimal number of qubits necessary for this task, namely four. This is achieved by optimizing the encoding of the data, using spatial and temporal multiplexing and recently developed read-out-schemes that also involve higher exponents of the reservoir response. We outline, test and validate our approach using eight prototypical three-dimensional chaotic systems. Both, the short-term prediction and the reproduction of the long-term system behavior (the system’s “climate”) are feasible with the same setup of optimized hyperparameters. Our results may be a further step towards the realization of a dedicated small quantum computer for prediction tasks in the NISQ-era.

## Introduction

A fundamental challenge in various disciplines of science, engineering, medicine, and economics is the prediction of complex dynamical systems^1^. The ability to predict future trends and behavior from historical data could lead to many advancements in the aforementioned fields. Recent progress in the field of data-driven artificial intelligence (AI) has led to great progress in many areas, including the forecasting of complex dynamical systems^2^. In this context reservoir computing (RC)^3–5^ has emerged as a well-suited approach to predict short- and long-term properties of chaotic dynamical systems^6–12^ that requires only small training datasets compared to other recurrent neural networks (RNNs), does not suffer from the vanishing gradient problem, and has small computational needs. At the core of the model is a random neural network with loops called reservoir that acts as a memory and yields a reservoir state to a given input. After initialization is the network topology fixed and only the weights of a linear output layer are optimized to map the reservoir state to the right output using linear regression. This practice of linearly mapping the reservoir response results in a fast and computationally efficient training. Apart from software-based reservoirs (so-called echo state networks - ESNs) exists the idea to realize a RC by a physical system, leading to novel, unconventional computers going beyond the capability of classical von Neumann computing concepts. A class of systems that are proposed for physical RC are controllable quantum systems - The exponentially large Hilbert space of a quantum system is supposed to be leveraged for time series forecasting. This branch of RC is called Quantum Reservoir Computing (QRC)^13–25^. QRC is motivated by advancements in the field of quantum computing and is a hybrid classical-quantum machine learning model. Due to the efficient, simple training, this framework is a good candidate for a quantum computing method that can outperform classical computing on NISQ era^26–28^ devices.

Here, we introduce a modified version of the basic QRC framework. Our novel approach combines elements from QRC already discussed in the literature (temporal^13,14^ and spatial multiplexing^15^), with state-of-the-art practices that stabilize and boost performance in ESNs, namely non-linear readout functions^29^ and a proper data preprocessing pipeline. The resulting novel framework is benchmarked with prototypical synthetic chaotic systems. The focus here is two-fold. The first goal is to statistically validate the short- and long-term forecasting results of QRC. This important step is - to the best of our knowledge - so far missing in current research. Secondly, we do want to showcase that the proposed framework is capable of achieving very good prediction results with extremely small simulated quantum systems. This is essential for the future application on NISQ devices. In Sect. 2 the general task, the simulation setup and the prediction results are presented. In Sect. 3 the results are discussed in the context of future applications and open questions are addressed. Finally, in Sect. 4 the model and its hyperparameter space, the details about the simulated quantum systems and the performance measures are introduced.

## Results

The modified version of the initially proposed QRC-algorithm^13,14^ investigated in this study is designed to forecast (continue) a *d*-dimensional discrete time series $\mathrm {\textbf{u}}(t)=\{\mathrm {\textbf{u}}_{j} \}_{j=1}^{L}$ $=\{\mathrm {\textbf{u}}(t_{0}),\mathrm {\textbf{u}}(t_{0}+\Delta t),\cdots \}$ from past time steps. Meaning, the model is supposed to approximate a function *f* that fulfils

1 $$\begin{aligned} \mathrm {\textbf{u}}_{k+1} = f (\{\mathrm {\textbf{u}}_{j} \}_{j=1}^{k}) \end{aligned}$$

and generalizes for unseen data. As mentioned in the introduction, RC approaches are well suited to solve such a task. Input data (in discrete time steps) are recurrently injected into the reservoir. The dynamics of the reservoir produces a high-dimensional and non-linear reservoir response that encodes information of the current state and the recent past of the dynamical system. The readout layer is trained to map this reservoir response (or readout vector) to the next step of the time series. To this end, QRC leverages the exponentially large Hilbert space of quantum systems (here multiple qubit systems), as an enhanced feature space. This is done in a way that the quantum state retains information about the present and past of the time series. The full algorithm is defined and explained in detail in Sect. 4.1. It follows a short description of the above described recurrent process. For each step *k* of the discrete time series, the current state of the time series is encoded into *r* quantum systems. The systems are then evolved by a unitary operator which scrambles the information. Thereafter, preselected expectation values are measured. This is done *V* times for each system before the next timstep $k+1$ is encoded into the systems. By collecting all these observables and also including higher exponents of these observables as additional nodes the output vector $\mathrm {\textbf{q}}(k)$ of step *k* (reservoir response of step *k*) is obtained. These vectors are trained by Ridge regression to linearly map the reservoir response onto the subsequent step of the time series and therefore fulfilling the above defined task.

### Simulation setup

In this study the prediction performance of the QRC model is investigated by forecasting eight different prototypical three-dimensional chaotic systems like the Lorenz-63 and the Rössler system (defined in Supplemental information S4) with a numerical simulation of small controllable quantum systems. To showcase the predictive power of the QRC model when very small qubits systems are accessible, all quantum systems are selected to be as small as the algorithm theoretically allows. Therefore all the results in this work are obtained with four qubit systems. Information regarding the simulated quantum system’s unitary evolution can be found in Sect. 4.3. The time series are preprocessed by standardization and subsequently scaled into the interval [*a*,*b*] with $0\le a<b\le 1$. This practice gives rise to two new hyperparameters: *a* and *b*. The measured expectation values that are selected to built the output vectors $\mathrm {\textbf{q}(k)}$ are the spin-projection $\langle \sigma _{\textrm{z}}^{i} \rangle$ and the spin-correlation $\langle \sigma _{\textrm{z}}^{i}\sigma _{\textrm{z}}^{l} \rangle$ with $i,l \in \{1,\ldots ,4\} \ \text {and} \ i<l$. The model has some free hyperparameters. The parameters that are part of the classical part of the algorithm are the scaling parameters *a* and *b*, the regression parameter $\mathrm {\beta }$ and the degree *G* of the used exponents of the reservoir readout. The free hyperparameters of this study concerning the quantum part of the algorithm are the number *r* of employed quantum systems and the number of employed evolution and measurement processes *V* per encoded time step *k*.

The results in this study are obtained for each investigated hyperparameter combination by evaluating the model for $N_{\textrm{stat}}$ times. For each of these runs, different parts of the chaotic attractors and different choices of the random parameters for the unitary transformations describing the quantum systems are selected to enable statistical significant performance evaluation. Each model is trained (see Sect. 4.1) with $N_{\textrm{sync}}+N_{\textrm{train}}=L$ consecutive steps of the trajectory and subsequently the time series is continued for $N_{\textrm{pred}}$ steps.

### Prediction results

In this work, we show that the introduced and simulated QRC model is capable of achieving short-term forecasting quality rivaling and in some cases outperforming “classical” RC methods and also accurately predicting the long-term climate of three-dimensional chaotic systems while utilizing four qubit systems and small training datasets $(N_{\textrm{sync}}=100$ and $N_{\textrm{train}}=2000$). Our main goal is to introduce techniques that make real world QRC-applications on near-term available quantum computers realistic. We choose a quantum system (unitary evolution) that works (more details Sect. 4.3) and do not further optimize it. The selected hyperparameters critically influence the prediction performance in a non-trivial way (see Supplemental information S1). Covering the hyperparameter space with a fine grid search to find the best performing hyperparameter combination is out of reach due to computational limitations. Instead, a Bayesian hyperparameter optimization using the python package Optuna^30^ over a hyperparameter space section is applied. For all eight investigated chaotic systems, the best performing configuration of hyperparameters is obtained by maximizing the mean forecast horizon (defined in Sect. 4.4) of the model ($N_{\textrm{stat}}$=100). With these best performing hyperparameter sets we train the model $N_{\textrm{stat}}$=500 times and forecast the trajectories for $N_{\textrm{pred}}$=20000 steps to evaluate the short- and long-term prediction efficiency of the model. The short-term prediction efficiency is measured by the forecast horizon, which determines the time for which the model prediction matches the actual continuation of the forecasted dynamical system up to a small deviation. The long-term prediction efficiency is determined by how well the model is able to recreate statistical properties of the attractor of the dynamical systems. Here we use as measures the largest Lyapunov exponent and correlation dimension. All the measures used to evaluate the prediction quality of these best performing models are defined in detail in Sect. 4.4. The inspected hyperparameter section and the best performing hyperparameter set for each chaotic system can be found in the Supplemental information (S2).

**Figure 1:**
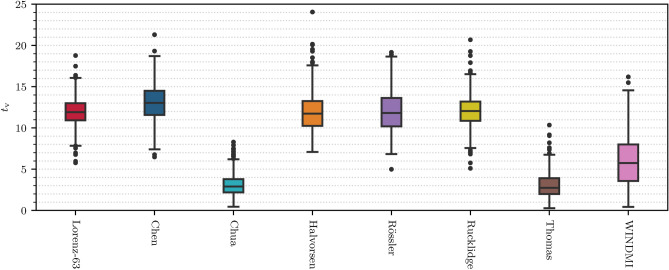
Distributions of the short-term accuracy for all eight forecasted chaotic systems. These are shown in a boxplot of the forecast horizon in Lyapunov times for the dynamical systems. The boxes represent the 25$\%$-75$\%$ percentile range of the data and the line in the middle of the box shows the median of the data, i.e. 50$\%$ of the forecast horizons are below this value. The extended lines showcase largest and smallest observation that falls within a distance of 1.5 times the interquartile distance (IQR) of the data. The black dots represent the outliers that are outside of this range. The mean and standard deviations of the distributions can be found in Table 1.

In many applications, it is important that the machine learning model is able to forecast the time series of a dynamical system very accurately for as long as possible. The best performing hyperparameter configurations are obtained by maximizing this ability. The mean forecast horizon of the 500 trained models can be found for each of the chaotic systems in Table 1 and the distributions are displayed as a boxplot in Fig.1. A comparison of our results with those of^31^ shows that the mean forecast horizon is in all cases at least comparably long as in the classical RC approach. Yet in some cases the QRC-models even outperform (larger mean forecast horizon) some hybrid RC approaches. This means that these simulated QRC models which are purely data-driven are able to forecast chaotic systems on longer time scales more accurately than some methods making use of prior knowledge about the physics of the underlying equations of the chaotic systems. This is especially true when the reservoir size is small in conventional hybrid RC. Investigating Table 1 and Fig. 1 shows that three (Chua, Thomas and WINDMI) of the eight chaotic systems are predicted accurately on a much shorter timescale than the other ones. Interestingly enough, these systems are also comparatively badly predicted with conventional RC. It is certainly an obvious and important question to figure out the causes for the systematic differences in performance among the model systems. Yet, this research topic is beyond the scope of this paper.

**Figure 2:**
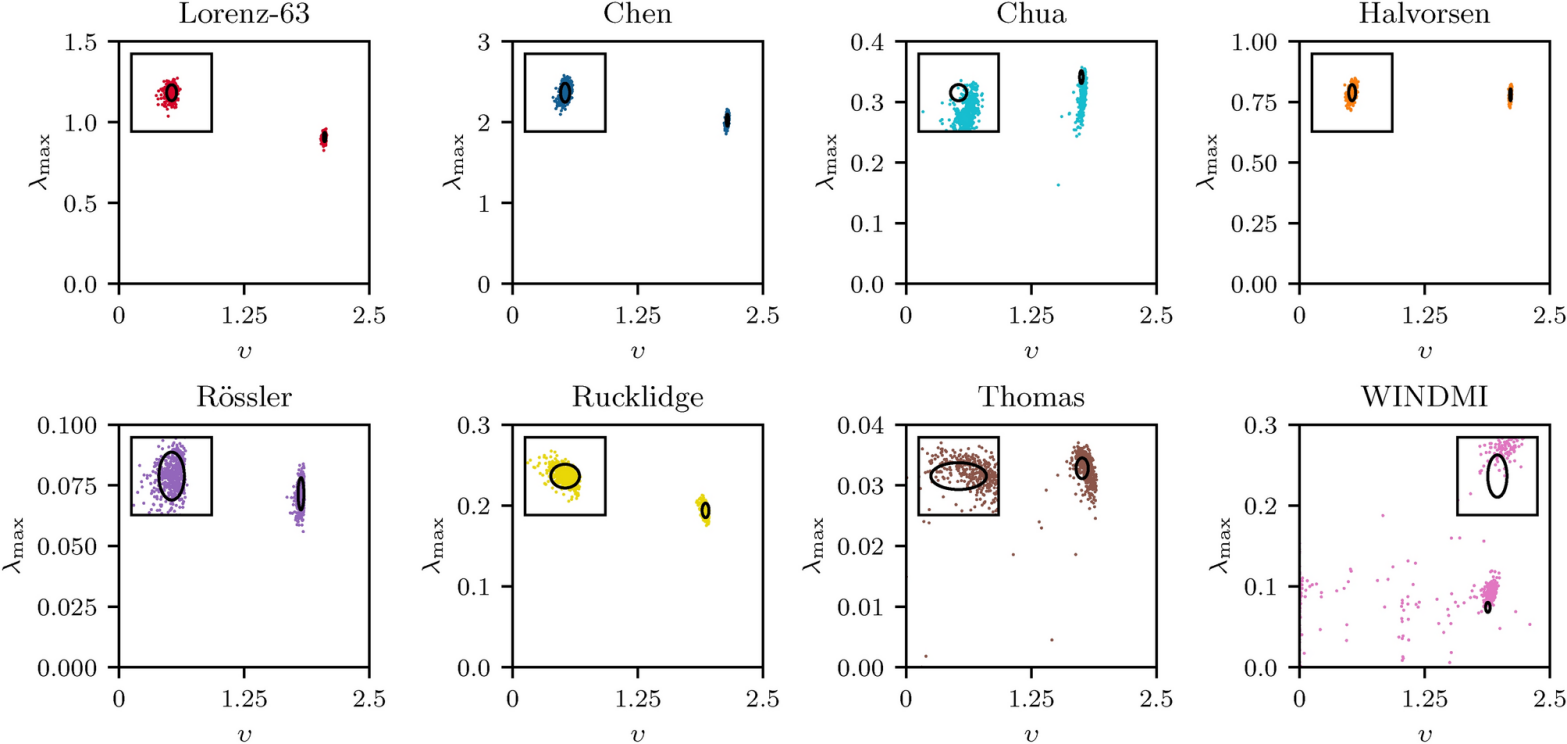
Distributions of the predicted climate for all eight forecasted chaotic systems. These are depicted by showing the Largest Lyapunov exponent vs correlation dimension for each of the 500 forecasted trajectories for all eight chaotic systems. The back ellipses show the three standard deviation errors of the correlation dimension and the largest Lyapunov exponent calculated from simulations of the actual systems. The zoomed-in windows plotted as insets are centered at $x= <\upsilon >_{true}$, $y = <\lambda _{\textrm{max}}>_{true}$) and extends $\pm 15 \% <\lambda _{\textrm{max}}>_{true}$ in the y-direction and $\pm 5\% <\upsilon >_{true}$ in the x-direction. The mean and standard deviations of the distributions can be found in Table 1.

In some other application scenarios, the focus might not be on the short-term behavior of the system, but rather on whether a dynamical system’s long-term properties (its “climate”) can be reproduced correctly. We investigate this by calculating two measures quantifying the (strange) attractors of the dynamical systems, namely the correlation dimension and the largest Lyapunov exponent (both defined in Sect. 4.4). For every chaotic system we use 500 trajectories, all consisting of 20000 steps that were not taken for training the models, to calculate the mean largest Lyapunov exponent and correlation dimension of the systems from true trajectories. We compare the calculated statistics of the attractors with the statistics of the forecasted time series. These findings are presented in Fig. 2 and the mean values are listed in Table 1. The spatial and temporal statistical properties of the five chaotic systems that are forecasted accurately for long time scales are extremely well predicted. In our sample, there are no single outliers with large deviations for the Lyapuov exponent or the correlation dimension. Rather, all realizations are within a $\approx 5 \sigma$ error range of the two measured quantities. For the remaining three systems, the climate of the systems is reproduced well in some cases, but some forecasted trajectories exhibit long-term behavior that is far from the statistical fluctuations of the true data. To the best of our knowledge, it is shown for the first time that QRC is capable to also reproduce the statistical long-term properties of predicted chaotic time series. These findings suggest that our (minimal) QRC setup does not only learn patters of the time series by heart leading to good short term predictions but rather correctly learns the dynamics of the underlying chaotic systems, enabling correct long term predictions.

**Table 1:** Table of mean values with standard deviation as derived from 500 realizations of all three evaluation measures for the chosen hyperparameter configuration of all eight chaotic systems. For comparison the results of the largest Lyapunov exponent and the correlation dimension for true trajectories are listed as well.

System	Mean $t_{v}$	Predicted mean $\lambda _{\textrm{max}}$	True mean $\lambda _{\textrm{max}}$	Predicted mean $\upsilon$	True mean $\upsilon$
Lorenz-63	11.9 ± 1.7	0.91 ± 0.02	0.91 ± 0.02	2.053 ± 0.008	2.052 ± 0.009
Chen	13.0 ± 2.2	2.02 ± 0.05	2.02 ± 0.05	2.146 ± 0.008	2.145 ± 0.008
Chua	3.1 ± 1.4	0.31 ± 0.02	0.341 ± 0.007	1.77 ± 0.02	1.75 ± 0.01
Halvorsen	11.9 ± 2.3	0.78 ± 0.02	0.78 ± 0.02	2.106 ± 0.006	2.106 ± 0.006
Rössler	11.9 ± 2.4	0.071 ± 0.005	0.072 ± 0.004	1.82 ± 0.02	1.82 ± 0.02
Rucklidge	12.1 ± 2.0	0.194 ± 0.006	0.194 ± 0.006	1.93 ± 0.02	1.93 ± 0.02
Thomas	3.0 ± 1.5	0.03 ± 0.004	0.033 ± 0.001	1.8 ± 0.2	1.76 ± 0.04
WINDMI	5.9 ± 2.9	0.08 ± 0.04	0.074 ± 0.004	1.5 ± 0.8	1.88 ± 0.02

## Discussion and conclusions

In this work, we present a QRC architecture that is suitable for small quantum systems and performs very well in forecasting low-dimensional chaotic dynamical systems. The eight systems can be predicted on at least a few Lyapunov times very accurately. We demonstrated for the first time that the model is also able to recreate the long term dynamics of the chaotic systems in most cases very well. A future goal should be to decrease the number and optimally fully remove those realizations in the three badly performing systems that completely diverge. First steps for such a procedure are sketched in the Supplemental information (S3). We find indications that the performance of the short term predictions and the correct reproduction of the long term properties of the system are related to the actual choice of random variables controlling the spin-spin interactions and the onsite disorder in the Ising model of the quantum reservoir. We want to highlight the fact that using multiple quantum systems in combination with multiple evolution and measurement processes is a key to achieve optimal performance with small quantum systems. All best performing hyperparameter configurations use the maximal number of reservoirs ($r=3$) of the range we selected for the Bayesian hyperparameter optimization. Furthermore, finding a good regression parameter is important for decreasing badly performing outliers. Our investigation of the hyperparameters a,b is very simplistic. Whether the performance can be increased by optimizing the hyperparameters over the continuous interval $0\le a<b\le 1$ is an open question. Other choices of how the time series is encoded might also help to achieve better forecasting ability of the models. Another open question is whether the performance can be further improved by increasing *G* or by using different readout functions $\mathrm {F_{res}}$. Other methods that are used to increase prediction performance in “classical” RC like adding noise to the training data to decrease overfitting and using other regression algorithms (e.g. tree-regression^32^) should also be investigated.

In conclusion, the results of our research in combination with more suitable unitary evolution for NISQ-devices could put hardware realizations and real-world applications of QRC not far out of reach. Future work should further investigate the employed unitary operator and look further into what defines a good performing quantum reservoir while ideally keeping NISQ-device restrictions (e.g. noise) in the hyperparameter and unitary operator selection in mind.

## Methods

### QRC framework

The input data is sequentially encoded for each step of the time series into the quantum system. For an *N* qubit system, this sequential input is realized by successively initializing the quantum state for each step of the time series into the state

2 $$\begin{aligned} {\rho _{k} = \rho _{{\mathrm {\textbf{u}^{1}}_{k}}} \otimes ...\otimes \rho _{{\mathrm {\textbf{u}}^{d}_{k}}} \otimes \textrm{Tr}_{1,\cdots ,d}(\rho (k-1)))} \end{aligned}$$

where $\rho _{{\textrm{u}^{i}_{k}}}=\left| {{\textrm{u}^{i}_{k}}}\right\rangle \!\left\langle {{\textrm{u}^{i}_{k}}}\right|$ and $\left| {{\textrm{u}^{i}_{k}}}\right\rangle =\sqrt{{1-\textrm{ u}^{i}_{k}}}\left| {0}\right\rangle +\sqrt{{\textrm{u}^{i}_{k}}}\left| {1}\right\rangle .$ Here and in the following, the *i*-th coefficient of the *k*-th step of the discrete time series $\mathrm {\textbf{u}}(t)$ is denoted as $\textrm{u}^{i}_{k}$ and $\textrm{Tr}_{1,\ldots ,d}(.)$ is the partial trace over the first *d* qubits. This encoding choice has two obvious consequences. The first one is that to retain information about past inputs, the number of qubits has to be larger than the dimension of the time series. The second one is that the time series has to be scaled to the interval [0,1]. Following the encoding of one time step the system evolves under unitary evolution. The full map between two quantum states of the sequence is

3 $$\begin{aligned} \rho (k) = \textrm{U} {\rho _{k}} \textrm{U}^{\dag }. \end{aligned}$$

Following the unitary evolution, expectation values are used to extract the high dimensional encoded information of the present and recent history of the time series. For each step of the time series, these expectation values (output nodes) are collected in the reservoir output vector $\mathrm {\textbf{n}}(k)$ of step *k*. The dimensionality of this vector is determined by the choice of observables and the number of qubits of the quantum system. One of the primary aims of this paper is to showcase the capacity QRC has even when only very small quantum systems are available. To increase the number of output nodes without increasing the number of qubits there are three methods employed.

*Temporal multiplexing*^13,14^ Rather than employing the unitary evolution and the measurement one time before encoding the next step of the time series, the evolution and measurement process is carried out *V* times. The output vectors for each of these single measurement phases are merged together into one output vector

4 $$\begin{aligned} \mathrm {\textbf{v}}(k)=\begin{pmatrix} \mathrm {\textbf{n}}_{1}(k) \\ \vdots \\ \mathrm {\textbf{n}}_{V}(k)\end{pmatrix} \end{aligned}$$

of step *k*.

*Spatial multiplexing*^15^ Rather than employing just one quantum system, multiple reservoirs are used, and the output vector of each of these reservoirs are concatenated together into one output vector of step *k*

5 $$\begin{aligned} \mathrm {\textbf{p}}(k)=\begin{pmatrix} \mathrm {\textbf{v}}_{1}(k) \\ \vdots \\ \mathrm {\textbf{v}}_{r}(k) \end{pmatrix}. \end{aligned}$$

*Reservoir readout function* The final technique to increase the dimension of the output vector is to apply a function $\mathrm {F_{res}}$. In this work, powers of the reservoir readout up to the fourth order are considered as the readout function. The studied choices are thus only the reservoir response (and a bias term)

6 $$\begin{aligned} \textbf{q}(k)=\mathrm {F_{res}}(\textbf{p}(k))=\mathrm {\begin{pmatrix} 1 \\ \textbf{p}(k) \end{pmatrix}}, \end{aligned}$$

the reservoir response and its square (also known as “Lu readout”^6^)

7 $$\begin{aligned} \mathrm {\textbf{q}}(k)=\mathrm {F_{res}}(\mathrm {\textbf{p}}(k))=\begin{pmatrix} 1 \\ \mathrm {\textbf{p}}(k) \\ \mathrm {\textbf{p}}^{2}(k) \end{pmatrix}, \end{aligned}$$

the reservoir response and its second and third power

8 $$\begin{aligned} \mathrm {\textbf{q}}(k)=\mathrm {F_{res}}(\mathrm {\textbf{p}}(k))=\begin{pmatrix} 1 \\ \mathrm {\textbf{p}}(k)\\ \mathrm {\textbf{p}}^{2}(k) \\ \mathrm {\textbf{p}}^{3}(k) \end{pmatrix}, \end{aligned}$$

and the reservoir response and its powers up to four

9 $$\begin{aligned} \mathrm {\textbf{q}}(k)=\mathrm {F_{res}}(\mathrm {\textbf{p}}(k))=\begin{pmatrix} 1 \\ \mathrm {\textbf{p}}(k)\\ \mathrm {\textbf{p}^{2}}(k) \\ \mathrm {\textbf{p}}^{3}(k)\\ \mathrm {\textbf{p}}^{4}(k)\end{pmatrix}. \end{aligned}$$

This selection of readout functions adds the hyperparameter *G* indicating the powers of the readout being included in the model. This method is inspired by new findings in “classical” RC, where it was shown that shifting the nonlinearities to the readout layer yields good prediction results even for minimal reservoirs^29^.

Hereafter, it is explained how these output vectors are exploited for time series forecasting.

*Training* The quantum systems are initialized in the quantum state

10 $$\begin{aligned} {\rho (0)}=(\left| {0}\right\rangle \langle {0}|)^{\otimes N}. \end{aligned}$$

For a given training data set $\{\mathrm {\textbf{u}}_{j} \} _{j=1}^{L}$ the first $N_{\textrm{sync}}$ steps are used to synchronize the quantum systems with the dynamics of the input data and thereby eliminate any transient dynamics that are a product of the initialization of the quantum states. The synchronization is accomplished by injecting the time series sequentially into the quantum reservoirs and letting the quantum states evolve as previously defined. The leftover $N_{\textrm{train}}$ steps of the training data set are used to obtain a readout matrix $\mathrm {\textbf{W}_{out}}$ such that the quantum reservoir output vector $\mathrm {\textbf{q}}(k)$ is mapped to step $k+1$ of time series $\mathrm {\textbf{u}}_{k+1}$. To do this, the system is sequentially injected with the remaining training data. The full output vectors $\mathrm {\textbf{q}}(k)$ are measured and collected in a matrix $\mathrm {\textbf{Q}}=[\mathrm {\textbf{q}}(N_{\textrm{sync}}+1),\ldots ,\mathrm {\textbf{q}}(N_{\textrm{sync}}+N_{\textrm{train}}-1) ]$ and the desired outputs are collected in a matrix $\mathrm {\textbf{Y}}=[\mathrm {\textbf{u}}_{N_{\textrm{sync}}+2},\ldots ,\mathrm {\textbf{u}}_{N_{\textrm{sync}}+N_{\textrm{train}}}]$. The objective is to find a matrix $\mathrm {\textbf{W}_{out}}$ that solves the equation

11 $$\begin{aligned} \mathrm {\textbf{Y}=\textbf{W}_{out}\textbf{Q}}. \end{aligned}$$

Ridge regression (detailed in Sect. 4.2) is used to obtain $\mathrm {\textbf{W}_{out}}$.

**Figure 3:**
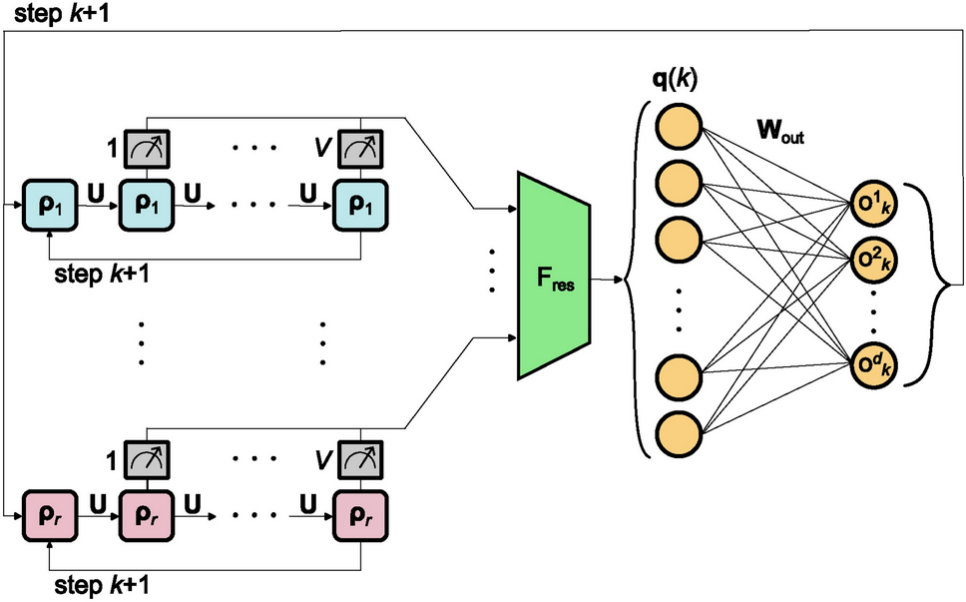
Schematic illustration of the prediction phase of the QRC model.

**Prediction**: Once the matrix $\mathrm {\textbf{W}_{out}}$ is obtained, arbitrary long predictions continuing the time series can be calculated. To continue the time series, an autonomously evolving closed-loop is employed. This is achieved by using the last prediction of the model

12 $$\begin{aligned} \mathrm {\textbf{o}}_{k+1}=\mathrm {\textbf{W}_{out}\textbf{q}}(k) \ \text {with} \ k>L-1. \end{aligned}$$

as the next input. The optimized readout matrix is kept fixed throughout the whole forecasting process. The prediction phase is schematically illustrated in Fig. 3. The continued time series is denoted as $\mathrm {\textbf{y}_{pred}}(t)=\{\mathrm {\textbf{o}}_{k}\}_{k=L+1}^{L+N_{\textrm{pred}}}$ in the following.

### Ridge regression

Ridge regression is used to obtain the readout matrix $\mathrm {\textbf{W}_{out}}$ by calculating

13 $$\begin{aligned} \textbf{W}_{\textrm{out}}=\textbf{Y}{\textbf{Q}^{\textrm{T}}}(\textbf{Q}\textbf{Q}^{\textrm{T}}-\beta \mathbbm {1})^{-1} \end{aligned}$$

which minimizes the error function

14 $$\begin{aligned} \sum _{N_{\textrm{sync}}<k<N_{\textrm{train}}} \Vert {\mathrm {\textbf{W}_{out}\textbf{q}}(k)-\mathrm {\textbf{u}}_{k}} \Vert ^{2}+\beta {{\textrm{Tr}}(\textbf{W}_{\textrm{out}}^{\textrm{T}}\textbf{W}_{\textrm{out}})}. \end{aligned}$$

$\mathrm {\beta }$ is an important hyperparameter that improves the prediction by penalizing large matrix coefficients.

### Simulation details: unitary operator

In this work, a spin network described by the transverse field Ising model plus onsite disorder

15 $$\begin{aligned} \textrm{H} = \sum _{i>j=1}^{N}J_{ij}\sigma _{\textrm{x}}^{i}\sigma _{\textrm{x}}^{j}+\frac{1}{2}\sum _{i=1}^{N}(h+D_{i})\sigma _{\textrm{z}}^{i} \end{aligned}$$

is chosen as the reservoir in alignment with previous QRC research^16^. In the equation above, *N* expresses the number of qubits and *h* is the magnetic field. The spin-spin couplings $J_{ij}$ are randomly selected from a uniform distribution in the interval [$-J/2, J/2$]. In the same manner, the onsite-disorders $D_{i}$ are randomly selected from the interval [$-W,W$]. Finally, $\sigma _{a}^{i}$ with $a \in$ $\{$x,y,z$\}$ denote the Pauli-matrices. The time evolution of the quantum system is utilized as the unitary operator. A unit time step size $\tau$ is chosen as the time between two consecutive inputs, and the observable measurements are carried out *V* times after letting the reservoirs evolve for a time $\tau /V$. The unitary operator that maps between states is

16 $$\begin{aligned} \textrm{U} = e^{\frac{-i\textrm{H}\tau }{V}}. \end{aligned}$$

Motivated by previous results^16^ the unit time step size $\tau =20J$ is not optimized and the quantum systems are chosen to be in the ergodic phase with $h=2/J$ and $W = 0.05/J$. All parameters are expressed in units of *J*. For convenience, *J*=1 is selected in the simulations. We described in Sect. 4.1 the model as general as possible because the choice of unitary operator is not unique. The appropriate unitary operator is going to be dependent on constraints of the available NISQ-devices. In^20^ unitary operator choices are investigated that might be more suitable for applications in the near-term future. The model introduced in our work is applicable to other unitary operators and therefore gives a good starting point for further research towards employing small quantum system for time series forecasting.

### Prediction performance measures

To evaluate the quality of the predictions, three different measures are used. These measures are chosen to sufficiently assess the quality of the exact short-term prediction and the reproduction of the long-term statistical properties (climate) of the systems. The measures used for the evaluation follow previous studies^33–35^ investigating “classical” RC.

The forecast horizon (also called valid time) is calculated to evaluate the short-term prediction capabilities of the model. It measures the time for which the continued time series matches very closely the true continuation of the trajectories. The forecast horizon is the elapsed time, while the normalized, time-dependent error *e*(*t*)^36^ between the continued time series $\mathrm {\textbf{y}_{pred}}(t)$ and the true continuation $\mathrm {\textbf{y}}(t)=\{\mathrm {\textbf{u}}_{k}\}_{k=L+1}^{L+N_{\textrm{pred}}}$ is smaller than a threshold value $e_{\textrm{max}}$. The normalized, time-dependent error is defined as

17 $$\begin{aligned} e(t)=\frac{\Vert \mathrm {\textbf{y}}(t) - \mathrm {\textbf{y}_{pred}}(t) \Vert }{\langle \Vert \mathrm {\textbf{y}}(t) \Vert ^{2}\rangle ^{1/2}}. \end{aligned}$$

Here, $\mathrm {\langle . \rangle }$ denotes the average over all $N_{\textrm{pred}}$ steps and $\Vert . \Vert$ is the L2-norm. It is determined for how many consecutive steps $s_{v}$ (starting with the first forecasted state) the relation $e(t)<e_{\textrm{max}}$ holds. In this work the threshold is chosen to be $e_{\textrm{max}}$=0.4. The forecast horizon $t_{v}$, in units of the Lyapunov times 1/$\lambda _{\textrm{max}}$ of the dynamical system, is obtained by calculating

18 $$\begin{aligned} t_{v}=\Delta t \cdot s_{v} \cdot \lambda _{\textrm{max}}. \end{aligned}$$

Here, $\Delta t$ is the time between two successive steps of the discretized time series, and $\lambda _{\textrm{max}}$ is the largest Lyapunov exponent (defined in the following paragraphs) of the system. The forecast horizon is measured in Lyapunov times to obtain a measure that is more comparable across different dynamical systems.

One aspect of the long-term behavior of a dynamical system is its structural complexity. The correlation dimension is a measure that quantifies the structural complexity by measuring the dimensionality of the space populated by the trajectory^37^. This measure is based on the discrete version of the correlation integral

19 $$\begin{aligned} C(r)=\lim \limits _{M \rightarrow \infty } \frac{1}{M^{2}} \sum _{i,j=1}^{M} \Theta (r - \Vert \mathrm {\textbf{x}}_{i}-\mathrm {\textbf{x}}_{j} \Vert ). \end{aligned}$$

Here, $\Theta$ represents the Heaviside function. *C*(*r*) calculates the mean probability that two states in phase space are closer than a threshold distance *r* for different time steps. For a self-similar strange attractor, the correlation dimension is defined by a power-law relationship in a certain range of the threshold *r*:

20 $$\begin{aligned} C(r)\propto r^{\upsilon }. \end{aligned}$$

The scaling exponent $\upsilon$ gives the correlation dimension of the attractor. The Grassberger Procasccia algorithm^38^ is used to calculate the correlation dimension from data.

Another characteristic of the long-term climate of a system is its temporal complexity. The most appropriate way to quantify the temporal complexity of a dynamical system is to analyze its Lyapunov exponents, characterizing the system’s development in time. A *d*-dimensional dynamical system has *d* Lyapunov exponents that determine the average rate of divergence of nearby points in phase space. By measuring the average rate of exponential growth of a small perturbation in each direction in phase space, the Lyapunov spectrum measures how sensitive the system is to its initial conditions. A dynamical system exhibits chaos if one of its Lyapunov exponents is positive, and the magnitude of the exponent determines the timescale on which the system becomes unpredictable^39,40^. The largest Lyapunov exponent $\lambda _{\textrm{max}}$ is linked to the direction in which the divergence occurs most rapidly,

21 $$\begin{aligned} d(t)=c \cdot e^{\lambda _{\textrm{max}}t}. \end{aligned}$$

In this research, measuring the largest Lyapunov exponent suffices, because of its dominant influence over the dynamics. This constraint also holds a computational advantage because $\lambda _{\textrm{max}}$ can be easily calculated from data using the Rosenstein algorithm^41^.

## Supplementary Information

Supplementary Information.

## Data Availability

The data that support the findings of this study are available from the corresponding author upon reasonable request.

## Code availability

The code used to generate the findings of this study is available from the corresponding author upon reasonable request.
