# Supplementary material for: Predicting three-dimensional chaotic systems with four qubit quantum systems
Source: Sci Rep. 2025 Feb 20;15:6201. doi: 10.1038/s41598-025-87768-0 (PMC11842815; doi:10.1038/s41598-025-87768-0)
Supplement: Supplementary file 1 — Supplementary Information. [file 41598_2025_87768_MOESM1_ESM.pdf]

Supplemental information
Predicting three-dimensional chaotic systems with four qubit quantum systems

Joel Steinegger[1,2] and Christoph Räth[1,†]

[1] Deutsches Zentrum für Luft- und Raumfahrt (DLR), Institut für KI Sicherheit, Wilhelm-Runge-Straße 10, 89081 Ulm, Germany
[2] Institut für Materialphysik im Weltraum, Deutsches Zentrum für Luft- und Raumfahrt (DLR), 51170 Köln, Germany
[†]Correspondence to: christoph.raeth@dlr.de

January 25, 2025

I. SUPPLEMENTARY NOTE 1: NON-TRIVIAL HYPERPARAMETERSPACE

In the following, we want to motivate the choice of free hyperparameters by showing the results of small and thoughtfully chosen parameter sweeps. They are obtained with N_{stat}=100 for each hyperparameter combination. In the original QRC algorithm [1] the Moore–Penrose-inverse is calculated to obtain the readout matrix. In this work, we use ridge regression instead of the pseudo inverse to counteract overfitting by adding a term that penalizes large matrix coefficients to the loss function. This is the common approach in "classical" RC. It introduces the regression parameter β that is critical to optimize. The importance of the introduction and optimization of β is analyzed by training the models for all $\beta \in \{10^{-25}, 10^{-24}, 10^{-23}, ..., 10^{-2}, 10^{-1}\}$ for 6 parameter configurations (see Table I). The obtained forecast horizons are depicted in Fig. 4 and the predicted climate is shown for $\beta \in \{10^{-1}, 10^{-3}, 10^{-10}, 10^{-20}\}$ in Fig. 8. Two important observations can be made. Firstly, it can be observed that good short- and long-term prediction quality depends critically on the right choice of β. Secondly, it can be seen that the importance of the optimization of the regression parameter seems to grow with the dimension of the output vector $\mathbf{q}(k)$. The first observation is further illustrated with the following example trajectories: Fig. 1 is an example of one of the prediction phases of the best performing hyperparameter combination (Table III) of the Lorenz-63 attractor presented for 1500 prediction steps. Changing the regression parameter to a very small one ($\beta = 10^{-24}$) frequently results in diverging trajectories, like in Fig. 2. A very large regression parameter ($\beta = 10$) typically leads to periodic predictions like in Fig. 3. These extreme parameter choices result in models that we can characterize as not working. This illustrates that the regression parameter has to be carefully optimized to

achieve good prediction performance. The increase of the reservoir output dimension and its effect on the short-term prediction quality are illustrated in Fig. 5 and Fig. 6 and its effect on the long-term climate is shown in Fig. 9 and Fig. 10. The forecasted continuation of the trajectories matches the actual continuation of the time series very closely for longer times when the dimension of the output vector $\mathbf{q}(k)$ is increased. The prediction of the climate patterns (especially the prediction of the largest Lyapunov exponent) increases with the output dimension of the output vector at step k. The remaining free hyperparameter is the scaling interval $[a,b]$. Its effects on the short- and long-term behavior are shown in Fig. 7 and Fig. 11. The right choice of $[a,b]$ seems to critically depend on the other hyperparameters.

configuration label	V	r	G	$[a,\, b]$	$\dim(\mathbf{q}(k))$
configuration 1	3	1	1	[0.10, 0.90]	31
configuration 2	4	2	3	[0.20, 0.80]	241
configuration 3	5	1	1	[0.20, 0.80]	51
configuration 4	5	1	2	[0.15, 0.85]	101
configuration 5	10	1	3	[0.10, 0.90]	301
configuration 6	4	1	4	[0.25, 0.75]	161

TABLE I. Table of all configurations V, G, r and $[a,\, b]$ and their labels used in the analysis of the influence of the regression parameter on the forecasting ability.

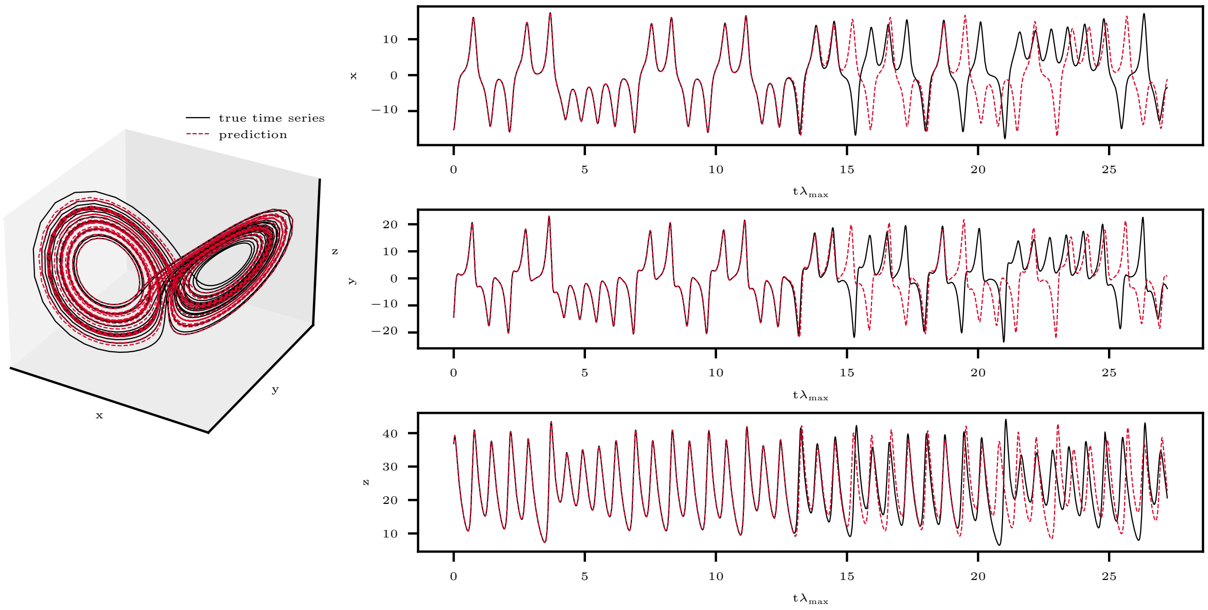

FIG. 1. Example of a trajectory of one of the 500 continued time series (TABLE III) obtained for the Lorenz attractor.

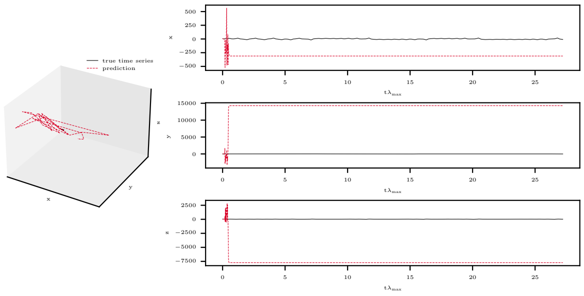

FIG. 2. Example of a trajectory of the prediction phase with a very small regression parameter.

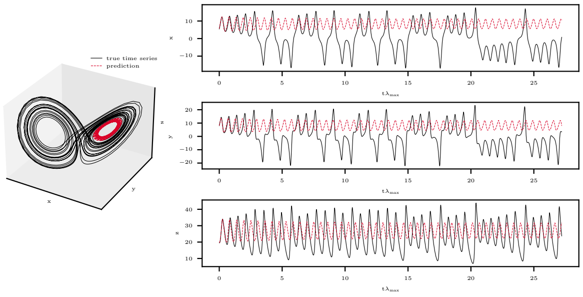

FIG. 3. Example of a trajectory of the prediction phase with a very large regression parameter.

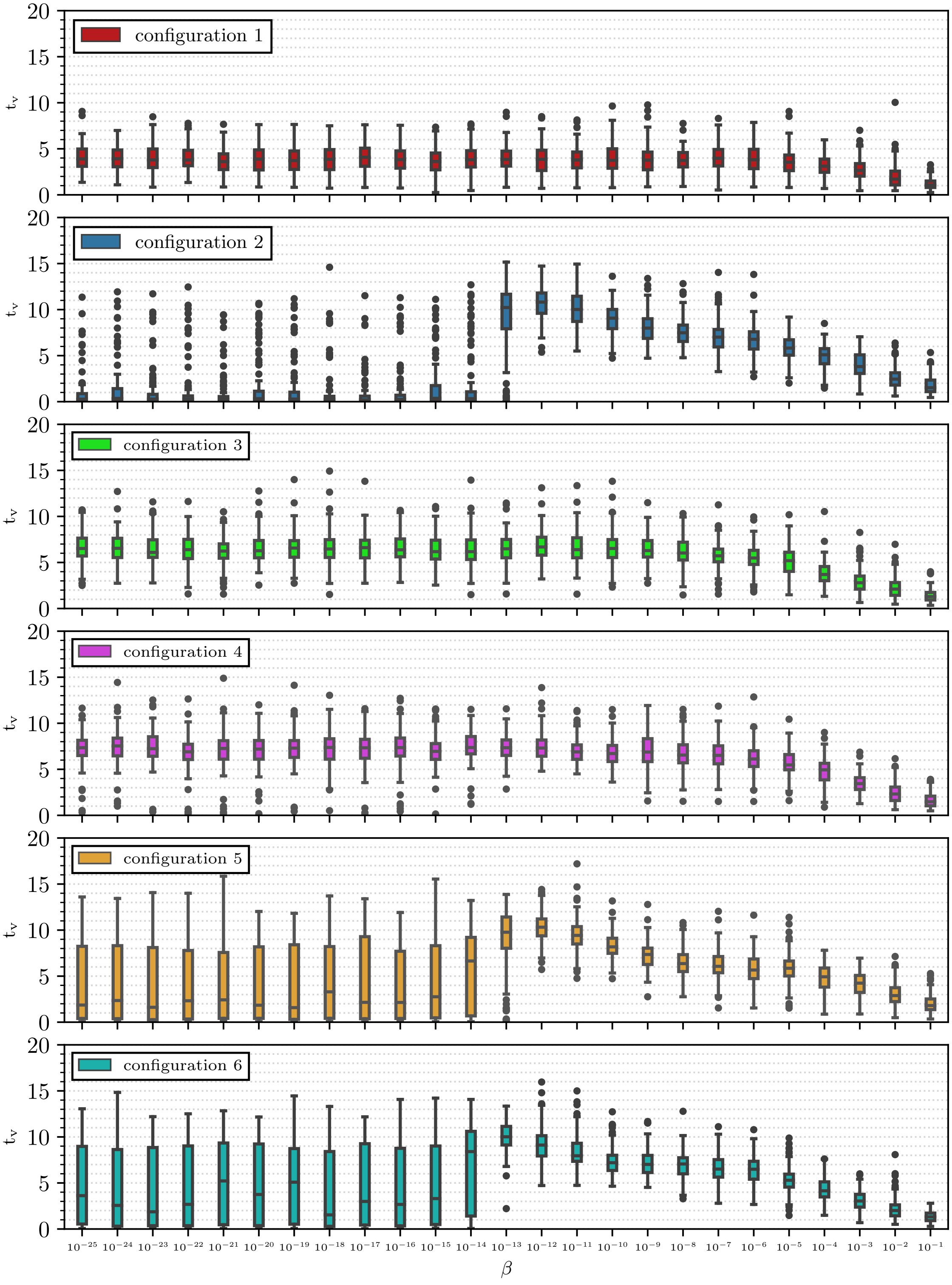

FIG. 4. Boxplot of the forecast horizon in Lyapunov times of 100 forecasted trajectories of the Lorenz-63 system against the regression parameter ($\beta \in \{10^{-1}, 10^{-3}, 10^{-10}, 10^{-20}\}$) for six different hyperparamter configurations of r, G, V and $[a, b]$ (see Table I).

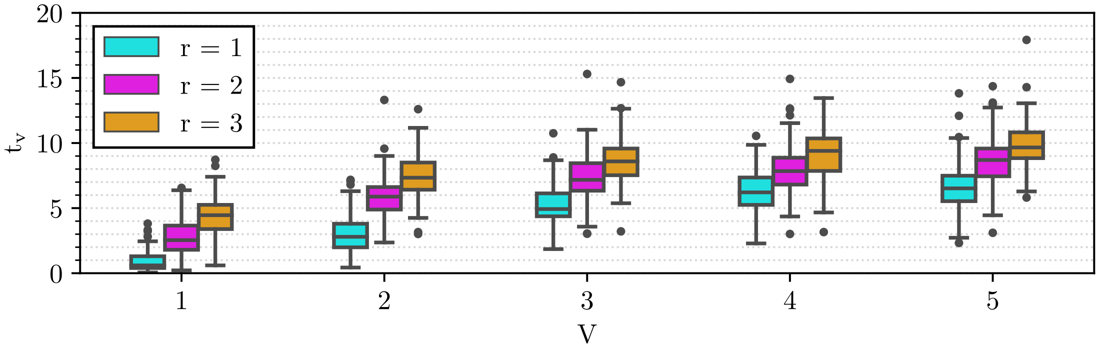

FIG. 5. Boxplot (100 forecasted trajectories) presenting the forecast horizon of the prediction of the Lorenz system with $[a, b]$=[0.2,0.8], $\beta = 10^{-10}$, $G = 1$ and varying V and r.

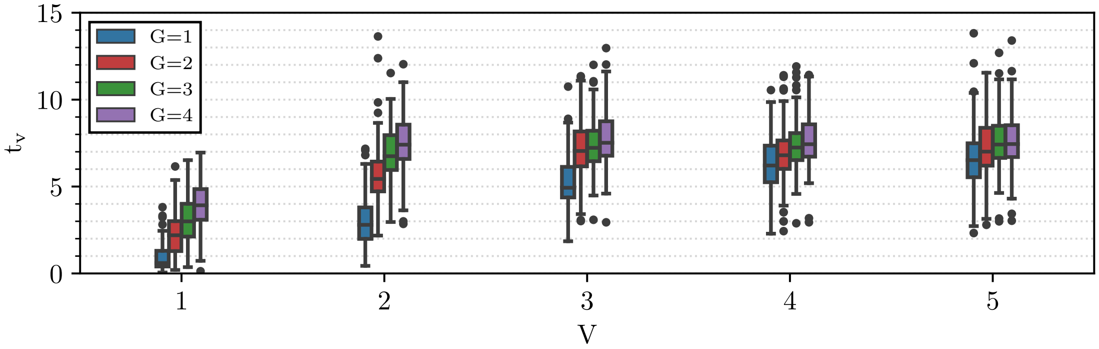

FIG. 6. Boxplot (100 forecasted trajectories) presenting the forecast horizon of the prediction of the Lorenz system with $[a, b]$=[0.2,0.8], $\beta = 10^{-10}$, r=1 and varying V and G.

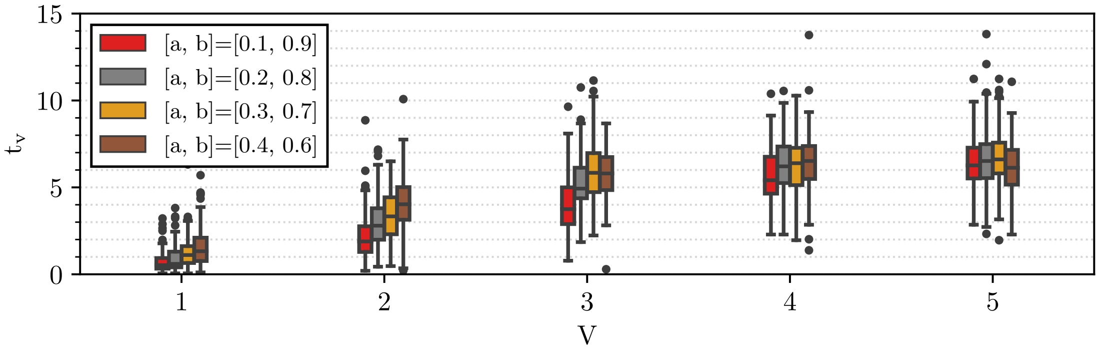

FIG. 7. Boxplot (100 forecasted trajectories) presenting the forecast horizon of the prediction of the Lorenz system with G=1, $\beta = 10^{-10}$, r=1 and varying V and $[a, b]$.

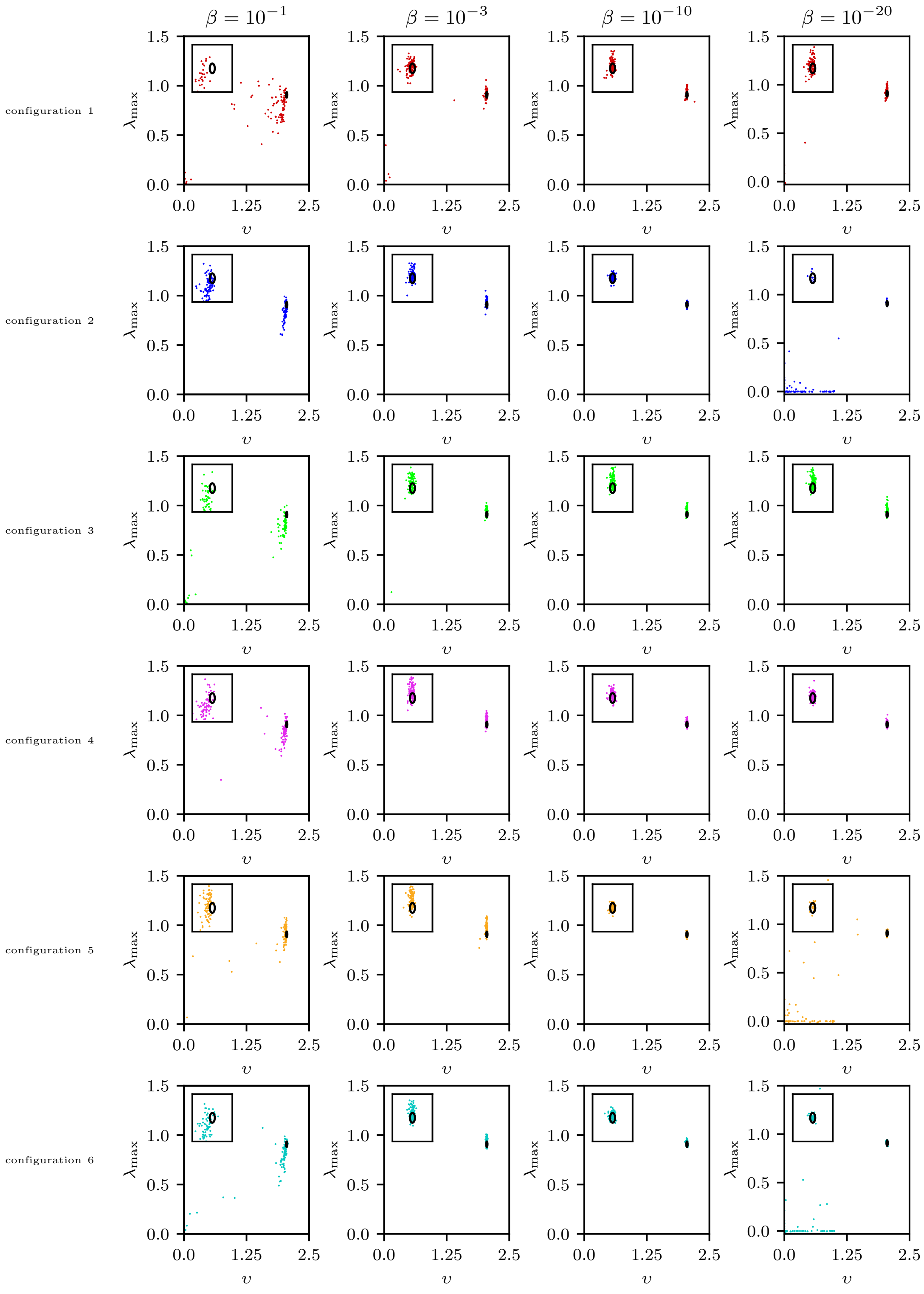

FIG. 8. Scatter plots of the predicted forecast horizons showing the predicted $\lambda_{\max}$ scattered against the predicted υ for each of the 100 realizations for $\beta \in \{10^{-1}, 10^{-3}, 10^{-10}, 10^{-20}\}$ and six different configurations of r, V, G and $[a, b]$ (see Table I).

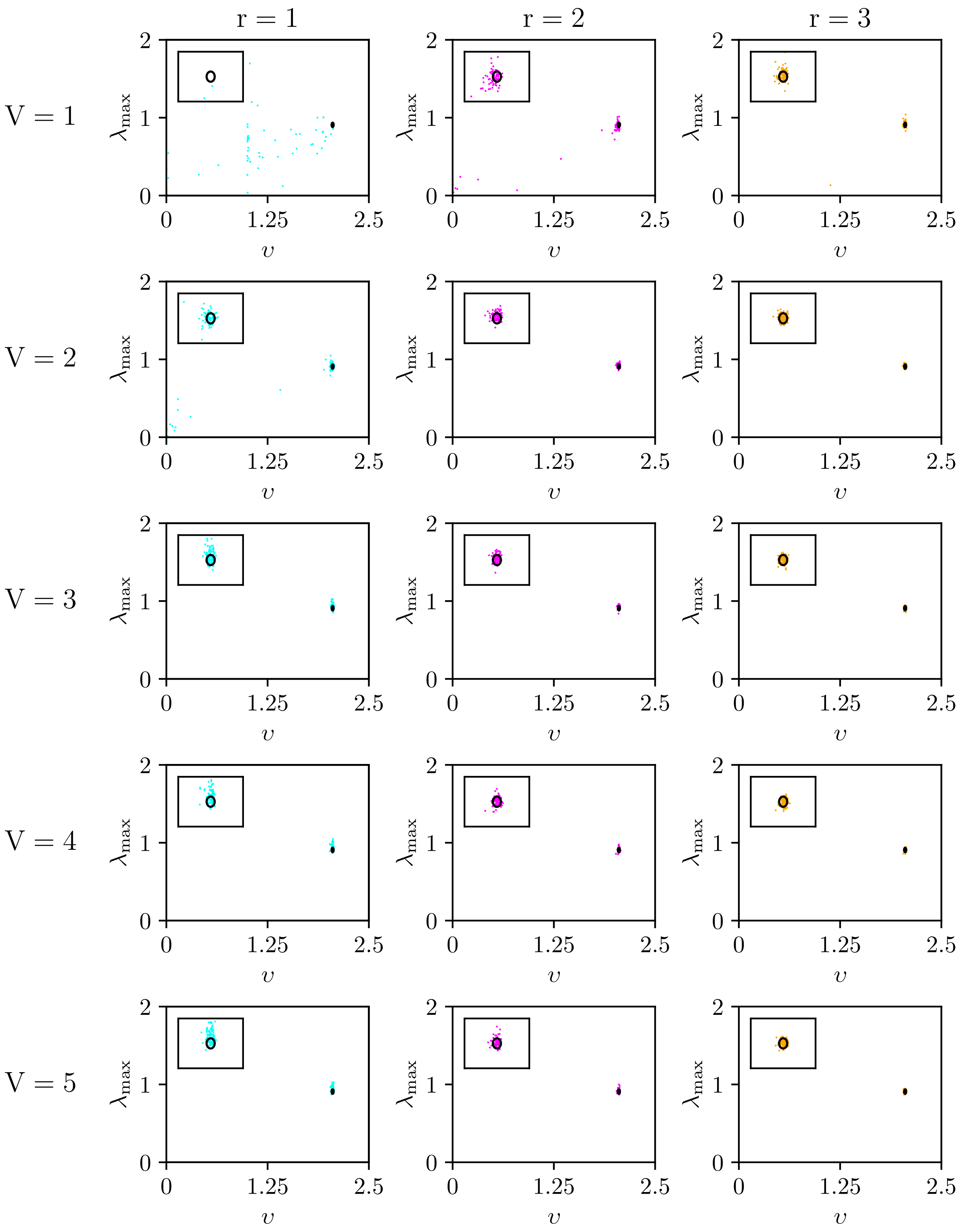

FIG. 9. Largest Lyapunov exponent scattered against correlation dimension 100 forecasted trajectories for each of the hyperparameter combinations varying V and r while holding [a,b]=[0.2,0.8], $\beta = 10^{-10}$ and $G = 1$ fixed.

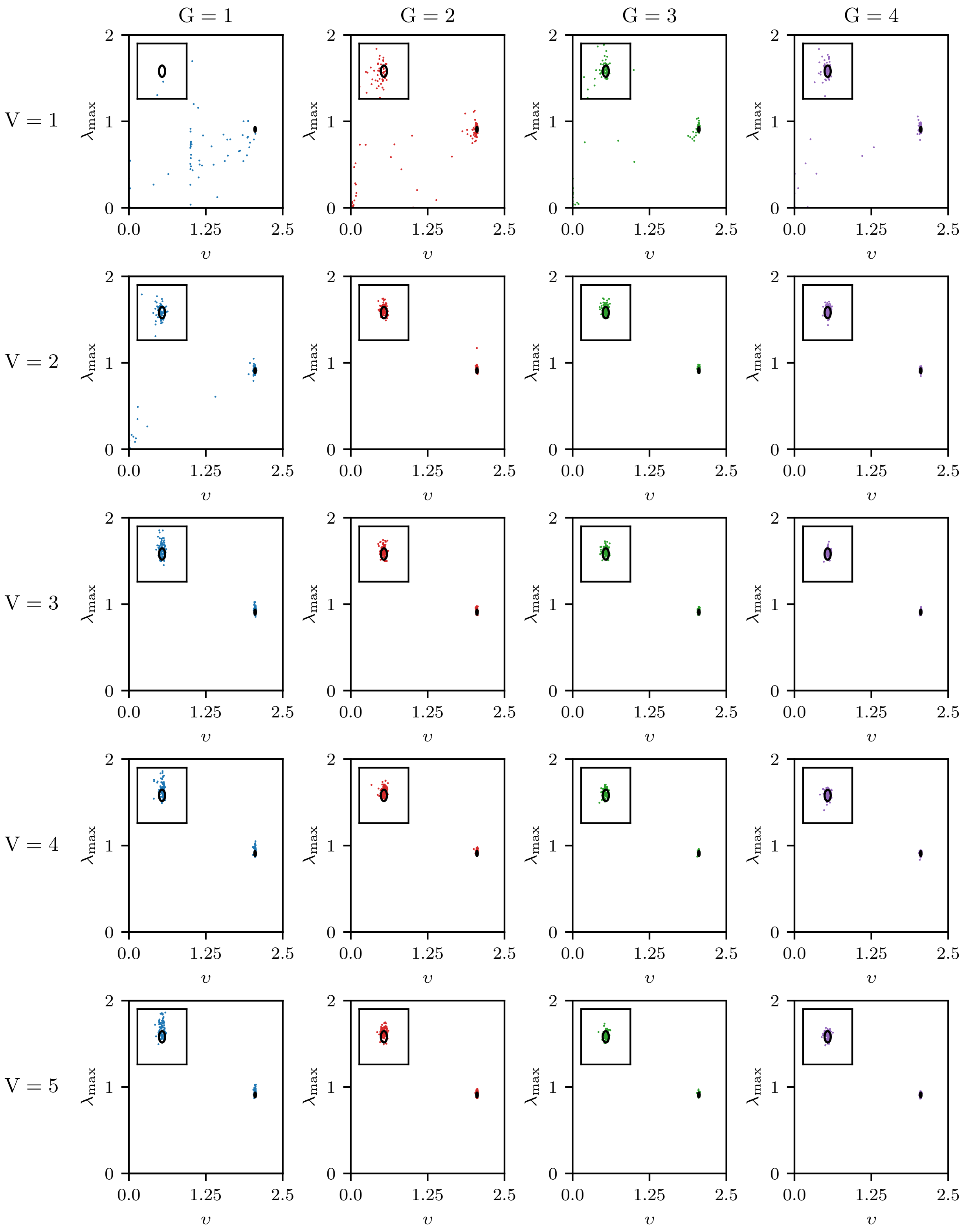

FIG. 10. Largest Lyapunov exponent scattered against correlation dimension 100 forecasted trajectories for each of the hyperparameter combinations varying V and G while holding [a,b]=[0.2,0.8], $\beta = 10^{-10}$, $r = 1$ fixed.

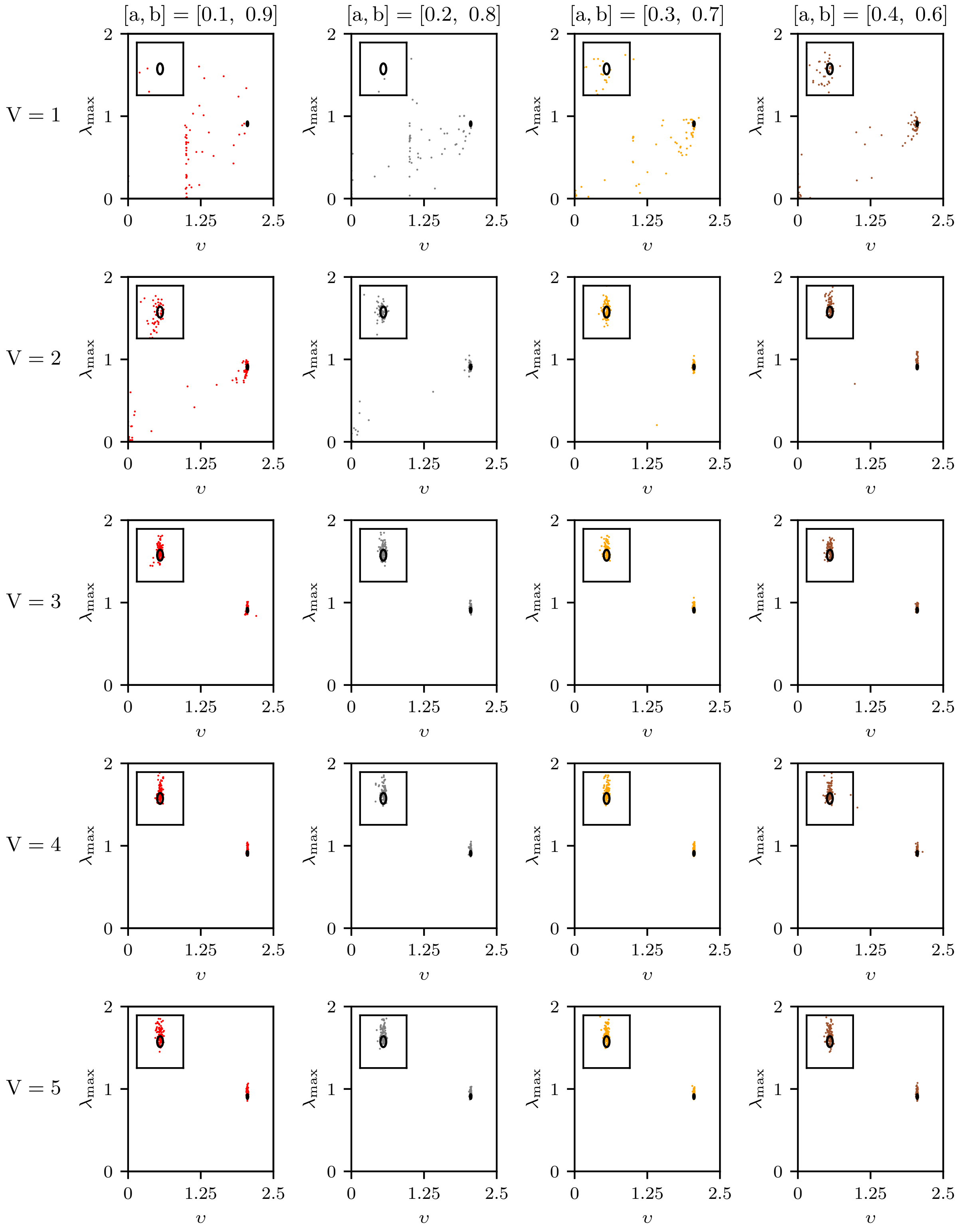

FIG. 11. Largest Lyapunov exponent scattered against correlation dimension 100 forecasted trajectories for each of the hyperparameter combinations varying varying V and $[a,b]$ while $G = 1$, $\beta = 10^{-10}$ and $r = 1$ fixed.

II. SUPPLEMENTARY NOTE 2: HYPERPARAMETERSPACE AND BEST PERFORMING HYPERPRAMETER CONFIGURATIONS

The hyperparameterspace that is examined by Bayesian hyperparameter optimization is displayed in Table II and we list the best performing hyperparameter sets that are used to obtain the results presented in main part of the paper, for each chaotic system in Table III.

Parameter	Parameter range
Number of evolutions:	$V \in \{1, 2, ..., 15\}$
Number of reservoirs:	$r \in \{1, 2, 3\}$
Regression parameter:	$10^{-20} < \beta < 10^{3}$
Readout polynominal:	$G \in \{1, 2, 3, 4\}$
Encoding interval:	$[a, b] \in \{[0.05, 0.95], [0.10, 0.90], [0.15, 0.85], \ldots, [0.40, 0.60], [0.45, 0.55]\}$

TABLE II. Hyperparameter space that is examined by the Bayesian hyperparameter search.

system	V	r	β	G	[a,b]
Lorenz-63	9	3	$1.41{\cdot}10^{-12}$	3	[0.15,0.85]
Chen	8	3	$1.09{\cdot}10^{-12}$	3	[0.30,0.70]
Chua	14	3	0.000269	2	[0.10,0.90]
Halvorsen	8	3	$1.41{\cdot}10^{-12}$	3	[0.20,0.80]
Rössler	9	3	$2.10{\cdot}10^{-12}$	3	[0.20,0.80]
Rucklidge	7	3	$1.25{\cdot}10^{-12}$	4	[0.15,0.85]
Thomas	15	3	$1.89\ {\cdot}10^{-10}$	4	[0.05,0.95]
WINDMI	10	3	$9.13{\cdot}10^{-12}$	4	[0.05,0.95]

TABLE III. Best performing Hyperparameter configuration for each chaotic system obtained by maximizing the forecast horizon with Bayesian hyperparameter optimization.

III. SUPPLEMENTARY NOTE 3: INVESTIGATION DIVERGING TRAJECTORIES (WINDMI)

We do not optimize the unitary evolution, i.e., the quantum reservoir, due to the reasons described in the main text. The type of unitary evolution (reservoir) and the parameter ranges are not changed in the optimization process. For a more complete optimization the pipeline introduced in this paper should also include the optimization of the unitary operators. This is clearly shown by the results of a small numerical experiment forecasting the WINDMI system, where the model's climate prediction is the worst. In Fig. 12 and Fig. 13 again the results of the best performing hyperparameter combination are presented together with results from a slightly changed experimental setup. For one trajectory the model is trained with the hyperparameters presented in III and therefore the unitary operators drawn from the uniform intervals until we find a model initialization with a forecast horizon that is larger than the mean forecast horizon of the best-performing model presented in the main part of the article. Afterward, 500 trajectories of the WINDMI system are trained and forecasted with exactly these unitary operators and hyperparameters. The long-term climate pattern of these results is presented in Fig. 12. The two plots clearly show that the unitary operator (”network connections”) has a huge impact on the prediction performance. The resulting mean forecast horizon is 8.1 ± 2.6 Lyapunov times. Hence, the short-term prediction quality is improved. The short-term prediction ability is further analyzed in Fig. 13. It is clear that using a ”well-performing” combination of unitary operators leads to the disappearance of the predicted trajectories that deviate after very short time scales from the real continuation of the time series, and also results in accurate climate prediction.

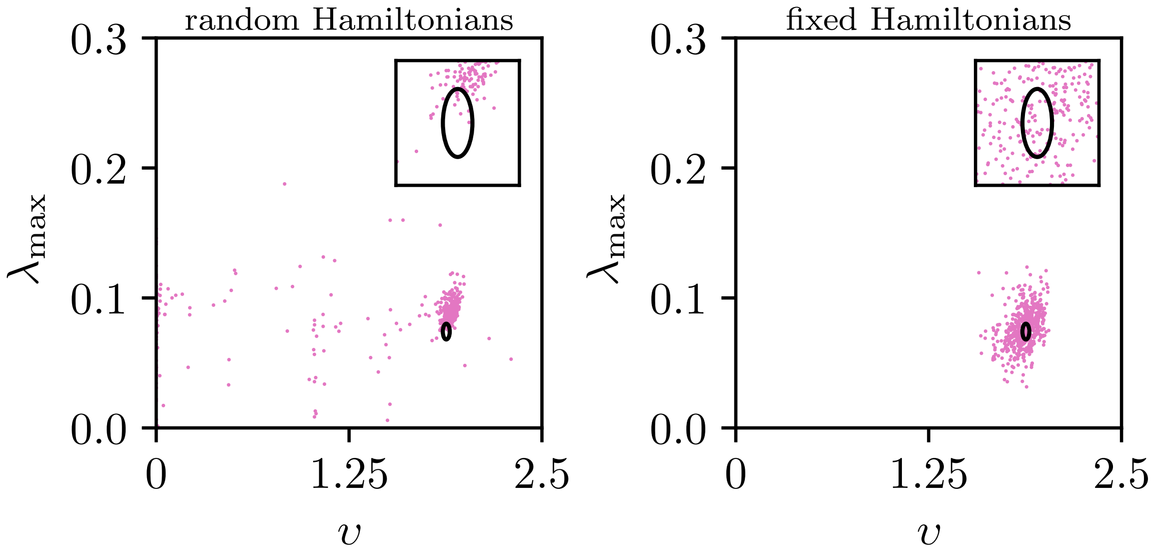

FIG. 12. Predicted climate for each of the 500 forecasted trajectories of the WINDMI system as presented in FIG. 2 (random Hamiltonians) in the main text and the same plot with fixed (well performing Hamiltonians) for the same hyperparameter configuration (see Table III).

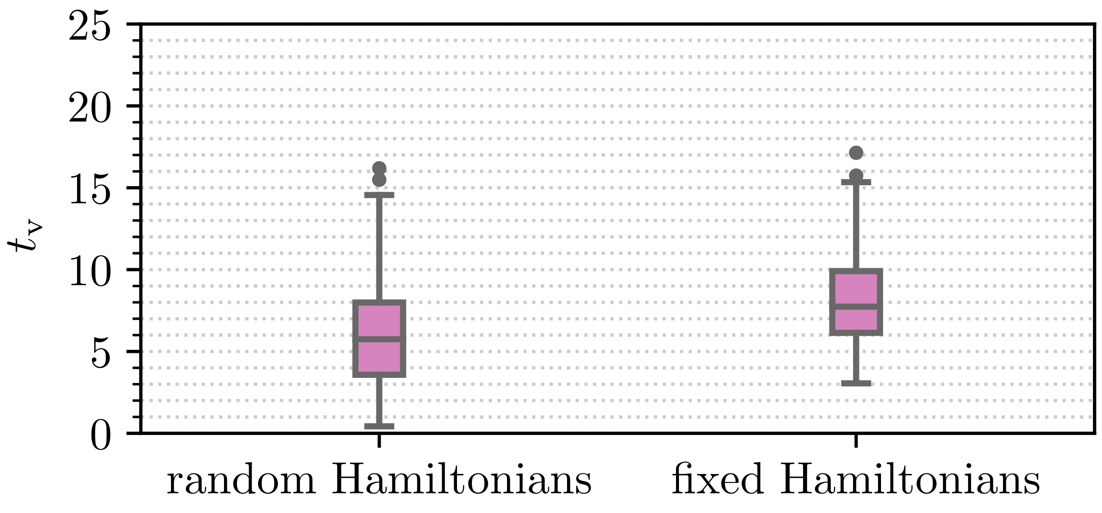

FIG. 13. Forecast horizon for randomly drawn Hamiltonians and fixed (well performing) Hamiltonians of the WINDMI system forecasting (500 trajectories each) with the hyperparameter configuration denoted in Table III.

IV. SUPPLEMENTARY NOTE 4: CHAOTIC SYSTEMS

Three-dimensional chaotic systems described by a flow F acting on the current state $\mathbf{u}(t)$ of the system

$$\dot{\mathbf{u}}(t) = F(\mathbf{u}(t)). \tag{1}$$

are used to display the potential QRC has in forecasting of nonlinear dynamical systems. The trajectories ($\mathbf{u}(t) = \{\mathbf{u}(t_0), \mathbf{u}(t_0 + \Delta t), \cdots\}$) are obtained by numerically solving Eq. 1 using the fourth-order Runge-Kutta method (RK4) [2–4]. In this study, eight three-dimensional prototypical chaotic systems with different system characteristics are tested. The differential equations describing the systems, the selected system parameter choices and details about the generation of the datasets are defined in the following paragraph.

The discrete time series used in our work are obtained employing the RK4 method with the starting point, Δt, and the system parameters chosen as specified below, to obtain 25001000 consecutive steps of the attractor. We throw away the first 1000 steps and split the remaining time series into 1000 different trajectories each consisting of 25000 steps. The system defining equations are listed in the following and the defining parameters are denoted in Table IV.

system	system parameters	Δt	initial state
Lorenz-63	$\rho = 28$, $\sigma = 10$, $\beta = 8/3$	0.02	[0, −0.01, 9]
Chen	$a = 35$, $b = 3$, $c = 28$	0.02	[−10, 0, 37]
Chua	$\alpha = 9$, $\beta = 100/7$, a = 8/7, b = 5/7	0.1	[0, 0, 0.6]
Halvorsen	$a = 1.27$	0.05	[−5, 0, 0]
Roessler	$a = b = 0.2, c = 5.7$	0.1	[−9, 0, 0]
Rucklidge	$\kappa = 2.0$, $\lambda = 6.7$	0.1	[1, 0, 4.5]
Thomas	$b = 0.18$	0.3	[0.1, 0, 0]
WINDMI	$a = 0.7, b = 2.5$	0.2	[0, 0.8, 0]

TABLE IV. System parameters, Δt and initial state for all eight chaotic systems that are forecasted in this work. The system parameters and the initial states are taken from [4].

Lorenz-63 :

$$\begin{aligned} \dot{x} &= \sigma(y - x) \\ \dot{y} &= x(\rho - z) - y \\ \dot{z} &= xy - \beta z \end{aligned} \tag{2}$$

Chen's system :

$$\begin{aligned} \dot{x} &= a(y - x) \\ \dot{y} &= (c - a)x - xz + cy \\ \dot{z} &= xy - bz \end{aligned} \tag{3}$$

Chua's circuit :

$$\begin{aligned} \dot{x} &= \alpha[y - x + bx + 0.5(a - b)(|x + 1| - |x - 1|)] \\ \dot{y} &= x - y + z \\ \dot{z} &= -\beta y \end{aligned} \tag{4}$$

Halvorsen attractor :

$$\begin{aligned} \dot{x} &= -ax - 4y - 4z - y^2 \\ \dot{y} &= -ay - 4z - 4x - z^2 \\ \dot{z} &= -az - 4x - 4y - x^2 \end{aligned} \tag{5}$$

Rössler attractor :

$$\begin{aligned} \dot{x} &= -y - z \\ \dot{y} &= x + ay \\ \dot{z} &= b + z(x - c) \end{aligned} \tag{6}$$

Rucklidge attractor :

$$\begin{aligned} \dot{x} &= -\kappa x + \lambda y - yz \\ \dot{y} &= x \\ \dot{z} &= -z + y^2 \end{aligned} \tag{7}$$

Thomas attractor :

$$\begin{aligned} \dot{x} &= -bx + \sin(y) \\ \dot{y} &= -by + \sin(z) \\ \dot{z} &= -bz + \sin(x) \end{aligned} \tag{8}$$

WINDMI attractor :

$$\begin{aligned} \dot{x} &= y \\ \dot{y} &= z \\ \dot{z} &= -az - y + b - \exp(x) \end{aligned} \tag{9}$$

V. SUPPLEMENTARY NOTE 5: NOISY QUANTUM SYSTEMS

The results of this work were obtained with the simulation of perfect quantum systems. We simulated noiseless unitary evolution of the quantum systems. For real world application this is not a realistic assumption because real quantum systems are not noiseless. One important step towards real world application is to investigate the effect of noise on QRC with the goal of finding unitary evolutions that can be implemented on real hardware, considering hardware limitations. This goes beyond the scope of the paper. Nevertheless we want to mention that there are first results showing that QRC seems to be quite resistant to noise and even benefit from a certain amount of noise in some cases [1, 5]. This gives hope that applications on NISQ era devices might be possible. Furthermore, some initial results

are presented that showcase that the described QRC framework using minimal amount (namely four) of qubits show a similar resistance against noise in the quantum reservoirs. The results are obtained by going from unitary evolution of the quantum reservoirs to evolutions obtained by solving the Lindblad equation numerically using the python package qutip [6]. As Lindblad operators, we consider for each qubit $L_i = \sqrt{\gamma}\sigma_z^i$. This describes a system with dephasing. We forecast the Lorenz system using one reservoir (r=1) for different noise levels (controlled by γ). For each noise level, we forecast $N_{\mathrm{stat}} = 10$ trajectories for N_{pred}=2000 steps. The forecasting results for each of these realizations are plotted in Fig. 14. One can observe that for a large range of γ, the prediction remains accurate on longer timescales compared to the noiseless case ($\gamma = 0$). As the noise increases further, the forecast horizon shortens, but the prediction does not completely break down.

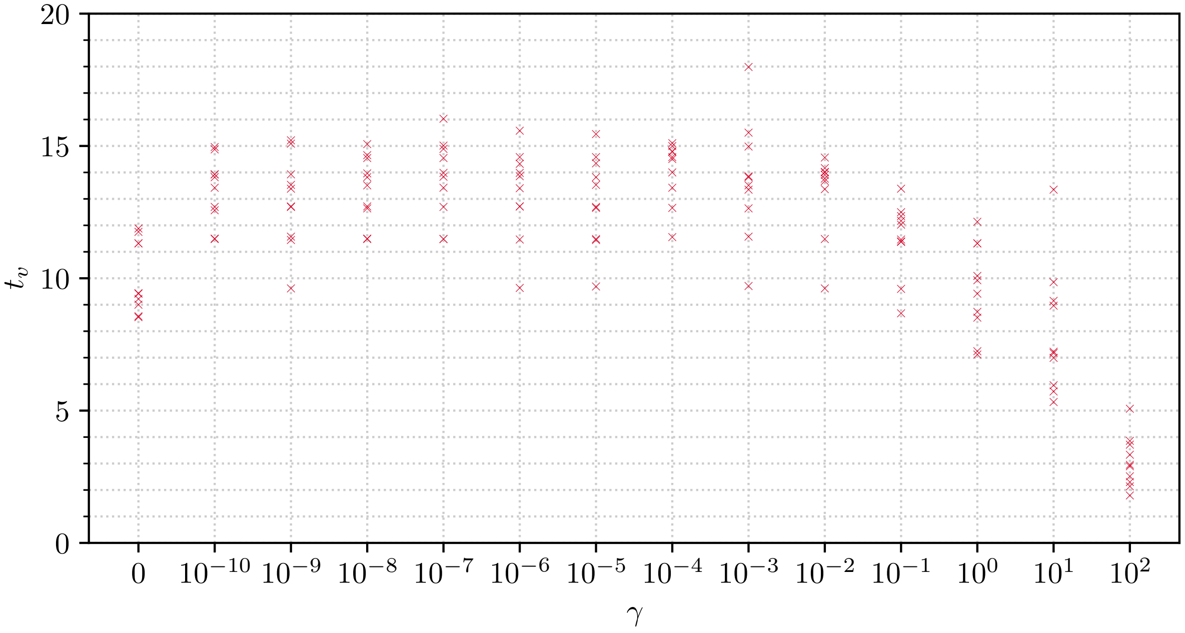

FIG. 14. Forecast horizon of the prediction of the Lorenz system obtained by noisy quantum reservoirs with $[a, b]$=[0.15,0.85], $\beta = 1.41 \times 10^{-12}$, $G = 4$ and $V = 9$ for various γ.

[1] K. Fujii and K. Nakajima, Harnessing disordered-ensemble quantum dynamics for machine learning, Physical Review Applied **8**, 10.1103/physrevapplied.8.024030 (2017).

[2] C. Runge, Ueber die numerische auflösung von differentialgleichungen., Mathematische Annalen **46**, 167 (1895).

[3] W. Kutta, Beitrag zur näherungsweisen Integration totaler Differentialgleichungen, Zeit. Math. Phys. **46**, 435 (1901).

[4] J. Sprott, *Chaos and Time-series Analysis* (Oxford University Press, 2003).

[5] L. Domingo, G. Carlo, and F. Borondo, Taking advantage of noise in quantum reservoir computing, Scientific Reports **13**, 10.1038/s41598-023-35461-5 (2023).

[6] J. Johansson, P. Nation, and F. Nori, Qutip 2: A python framework for the dynamics of open quantum systems, Computer Physics Communications **184**, 1234 (2013).
